# Morphine-induced *μ*-opioid receptor isoform MOR-1X amplifies dependence-related signaling

**DOI:** 10.3389/fnins.2026.1907881

**Published:** 2026-08-19

**Authors:** Michael Swingler, Martina Donadoni, Senem Cakir, Muhammed Bishir, Wenfei Huang, Sulie L. Chang, Ilker K. Sariyer

**Affiliations:** 1 Department of Microbiology, Immunology, and Inflammation, Center for Neurovirology and Gene Editing, Temple University Lewis Katz School of Medicine, Philadelphia, PA, United States; 2 Institute of NeuroImmune Pharmacology, Seton Hall University, South Orange, NJ, United States

**Keywords:** alternative splicing, cerebral organoids, dependence, IPA analysis, MOR-1, MOR-1X, opioids, OPRM1

## Abstract

Clinically used opioids, such as morphine, primarily act through the *μ*-opioid receptor (MOR), encoded by the *OPRM1* gene, which undergoes extensive alternative splicing. More than 20 *OPRM1* isoforms have been identified, yet functional characterization has largely focused on the canonical MOR-1 variant. With the emergence of three-dimensional human cerebral organoids (hCOs) derived from induced pluripotent stem cells (iPSCs), it is now possible to model human-specific neuronal responses to opioids more accurately. In this study, we established hCOs as a functional platform to investigate the impact of morphine on *OPRM1* pre-mRNA splicing and opioid signaling. We generated iPSC-derived hCOs and neurons, after which they were treated with or without morphine and screened using cellular and molecular/biochemical assays. Our results revealed that morphine exposure selectively induced the MOR-1X isoform in hCOs and iPSC-derived neurons in a dose-dependent manner as revealed by RT-PCR and RT-qPCR. We utilized CRE/CREB overexpression plasmid and lentiviral constructs to measure effects of morphine on cyclic AMP (cAMP) signaling. Upon morphine withdrawal, cells expressing MOR-1X exhibited markedly enhanced cAMP superactivation, a molecular hallmark of opioid dependence, compared with MOR-1. Furthermore, isoform-specific knockdown of MOR-1X by a short hairpin RNA (shRNA) effectively abolished this cAMP overshoot in iPSC-derived neurons. Collectively, these findings identify MOR-1X as a morphine-inducible isoform with a potential key role in the molecular mechanisms underlying opioid signaling, adaptation, and dependence in the brain.

## 1 Introduction

It is estimated that 1 in 50 people over the age of 12 in the USA have an opioid use disorder (OUD) (Dowell, 2024; CDC, 2025). Both clinically and illicitly used opioids act primarily on the *μ*-opioid receptor (MOR), which is encoded by the human *OPRM1* gene (Valentino and Volkow, 2018; Herman et al., 2024). Many studies have revealed that the human *OPRM1* gene undergoes extensive alternative splicing, with over 20 isoforms being identified from a single gene (Pan et al., 1999, 2005; Pasternak et al., 2020; Abrimian et al., 2021). The MOR isoforms can be broken down into full-length 7 transmembrane isoforms (7TM), truncated 6TM isoforms, or further truncated 1TM isoforms (Liu et al., 2021; Piltonen et al., 2021; Swingler et al., 2025). Differential binding affinity to a variety of substrates has been identified in these isoforms (Pan et al., 1999, 2005). Furthermore, these receptors appear to have different rates of GTP binding, internalization, and desensitization in response to different ligands (Koch et al., 2001; Gris et al., 2010). The full-length isoforms of MOR are known to have different C-terminal tails, which is the main site of phosphorylation upon activation by an opioid (El Kouhen et al., 2001; Chakrabarti et al., 2020). Thus, there is the possibility that these isoforms may activate different signaling cascades from each other. However, only the MOR-1 isoform has been extensively studied. As a result, little information is known about the activity of other isoforms and their function in response to agonism.

We have previously shown that morphine preferentially induces the expression of alternatively spliced isoform MOR-1X over MOR-1 in neuroblastoma cell lines (Regan et al., 2016; Donadoni et al., 2022). MOR-1X contains a unique exon, exon X, that is not present in any of the other isoforms. This exon results in a C-terminal domain that is substantially longer than MOR-1 and contains additional phosphorylation sites. Functional kinase arrays revealed unique signaling of MOR-1X in the ERK and p90 RSK pathways when compared to MOR-1 (Regan et al., 2016). Furthermore, Brown et al. has found that MOR-1X is upregulated in post-mortem brain tissue obtained from heroin users (Brown et al., 2022). Together, this data suggests that MOR-1X is inducible by exogenous opioids and may have different functional consequences than MOR-1. However, despite these observations, the differences between MOR-1X and MOR-1 in opioid signaling, adaptation, and dependence remain unknown.

MOR-1X remains a uniquely human isoform. As a result, *in vitro* models are necessary for the study of human specific OPRM1 splicing and the downstream effects of specific isoforms. Human cerebral organoids (hCOs) provide a unique model for studying human-specific aspects of brain development and disease (Lu et al., 2022; Birtele et al., 2025). hCOs are three-dimensional (3D) cell culture structures generated from pluripotent stem cells (hPSCs) that recapitulates aspects of the human brain. These 3D structures possess neuronal cell types and regional organization mimicking that of a human brain (Lancaster et al., 2013; Zhao et al., 2022; Donadoni et al., 2024). Recent studies have demonstrated that hCOs can recapitulate aspects of opioid exposure seen *in vivo*, including altered neuronal maturation, synaptic signaling, and stress pathway activation (Fernandes et al., 2022; Ho et al., 2024; Kim et al., 2024). Chronic opioid treatment of hCOs leads to measurable changes in neuronal excitability and gene expression patterns consistent with tolerance and dependence, including dysregulation of cyclic AMP (cAMP) signaling, cAMP response element binding protein (CREB) activation, and stress-response pathways (Kim et al., 2024). cAMP signaling and CREB activation are crucial pathways for studying opioids *in vitro*, as the inhibition and superaction of the cAMP pathway are the standards in measuring cellular levels of opioid dependence (Sharma et al., 1975b, 1975a, 1977; Williams et al., 2013; Muntean et al., 2019; Vandeputte et al., 2022; Klein et al., 2026).

Here, we explored the potential of hCOs as a model for studying opioid signaling and dependence, focusing specifically on the induction and signaling behavior of the MOR-1X isoform. We characterized MOR-1X expression dynamics in hCOs following opioid exposure and characterized MOR-1X specific effects on physiological dependence *in vitro*. Our findings aim to not only establish hCOs as a model of opioid signaling, but to shed light on isoform-specific mechanisms contributing to opioid dependence and tolerance in human neural systems.

## 2 Materials and methods

### 2.1 2D and 3D cell cultures

We generated human induced pluripotent stem cell (hiPSC) derived human cerebral organoids (hCOs) using STEMdiff™ Cerebral Organoid Kit (STEMCELL TECHNOLOGIES, 08570), and following manufacturer’s instructions with few modifications as we previously described (Bellizzi et al., 2024; Donadoni et al., 2024). Briefly, hiPSCs were seeded in 96-Well Ultra-Low Attachment Round-Bottom plates (Millipore Sigma, CLS7007) at a density of 12,000 cells/well in embryoid body (EB) seeding media and cultured for 6 days. On day 6, each EB was transferred into a single well of 24-Well Ultra-Low Attachment plates (Millipore Sigma, CLS3473) containing Induction Medium. On day 8, each EB was embedded in 15 μL of Matrigel^®^ (CORNING, 354277) on the organoid embedding sheets (STEMCELL TECHNOLOGIES, 08579) and transferred to 6-Well Ultra-Low Attachment plates (STEMCELL TECHNOLOGIES, 38071) containing expansion media. On day 12, EBs were switched to maturation media and placed on an orbital shaker and incubated at 37 °C with 5% CO_2_. A half-media change was performed every 3 to 4 days during and after maturation with identical conditions. hCOs were considered mature 50 days after seeding for EBs.

hiPSCs were differentiated into neural progenitor cells (NPCs) using STEMdiff™ SMADi Neural Induction Kit (STEMCELL TECHNOLOGIES, 08581). After 3 passages in induction media, cells are considered NPCs and are cultured in STEMdiff™ Neural Progenitor Medium (STEMCELL TECHNOLOGIES, 05833). These NPCs were differentiated into forebrain or midbrain neurons using STEMdiff™ Forebrain Neuron Differentiation and Maturation Kits (STEMCELL TECHNOLOGIES, 08600 and 08605), or STEMdiff™ Midbrain Neuron Differentiation and Maturation Kits (STEMCELL TECHNOLOGIES, 100–0038 and 100–0041) respectively. NPCs were cultured in differentiation media for 1 week with daily media changes. Following differentiation, cells were cultured for 2 weeks in maturation media, with media changes every 2–3 days, after which they were considered mature neurons. Experiments were carried out within 1 week of neurons reaching maturation. Neurons were incubated at 37 °C with 5% CO_2_.

HEK293 and HEK293T cells, human kidney cell lines, were obtained from ATCC. Cells were cultured in Dulbecco’s Modified Eagle Medium (DMEM) with 10% fetal bovine serum (FBS) and 0.3% gentamicin. Cells were maintained on poly-D-lysine coated flasks at 37 °C with 5% CO_2_.

### 2.2 Morphine treatments

Morphine (Sigma-Aldrich, M8777-50MG) was reconstituted in molecular grade H_2_O at a concentration of 10 mM and frozen in single use aliquots at −80 °C. Morphine was diluted to working concentration and added to culture system to reach the desired final concentration. hCOs and neurons were treated with 5 μM morphine to measure cAMP activity in an acute and withdrawal paradigm, as well as screening for MOR isoform induction. hCOs were also subjected to 0.5 μM, 1 μM, and 5 μM of morphine for 6 h to measure induction of MOR-1X in a dose dependent manner. iPSC-derived neurons were treated with the same dose response concentrations. HEK293 cells were treated with 1 μM morphine to measure cAMP activity and receptor response. Morphine doses were selected to fall within clinically relevant translatable ranges. Naloxone hydrochloride dihydrate was obtained from Sigma (Sigma-Aldrich, PHR1802).

### 2.3 hCOs and cell models of cAMP assays

To look at the effects of acute and chronic morphine treatment on cAMP signaling *in vitro*, we utilized a CRE/CREB Luciferase Reporter Lentivirus (BPS biosciences, 79,580) or pGL4.29[luc2P/CRE/Hygro] vector (Promega, 9PIE847) which contain a cAMP response element (CRE) that drives the transcription of the luciferase reporter gene luc2P. Increased/decreased levels of cAMP within the cell will result in increase/decrease in luciferase activity respectively, therefore a change in luminescence which corresponds to cAMP levels. All cAMP experiments were treated with a stimulation mixture (IBMX 0.5 mM, Ro20-1724 0.5 mM, forskolin 0.5 μM or 1 μM for cells or hCOs) for 4-h to induce cAMP to a readable level (Xia et al., 2011). 4-h was used for all acute treatments. 18-h post morphine was used for chronic treatment in HEK293 cells based on protocol established by Xia et al. (2011). Timing for chronic treatment in hCOs and neurons was optimized for dependence formation. hCOs were transduced with 5 MOI (multiplicity of infection) CRE-lentivirus using TransDux™ MAX Lentivirus Transduction Reagent (System Biosciences, LV860A-1). 72 h after transduction, hCOs were treated with stimulation mixture with or without 5 μM morphine for 4 h to measure acute inhibition of cAMP. To measure the effect of morphine withdrawal on hCOs, hCOs were treated twice with 5 μM of morphine 48- and 72-h after transduction. 18 h after the second morphine treatment, hCOs were washed 3X with PBS to remove morphine and initiate withdrawal. hCOs in both acute and chronic experiments were transferred to white walled, optical bottom 96-well plates (Thermo Scientific™, 165,306) before stimulation mixture was added. Forebrain neural precursors were plated at 20 k cells/well in 96-well plate. Following 2-weeks of maturation, cells were co-transduced with CRE-lentivirus and lentivirus expressing either a scrambled short-hairpin RNA (shRNA) or a shRNA specific for MOR-1X isoform, produced from the lentiviral expression vector pLKO.1. Following transduction, cells were treated with acute and chronic morphine using the same timeline as hCOs listed above. HEK293 cells were co-transfected with the pGL4.29[luc2P/CRE/Hygro] CRE plasmid (0.1 μg/well) and pcDNA3.1 plasmids expressing either MOR-1 or MOR-1X (5 ng/well) in Poly-D-Lysine pretreated 96-well plates (Corning, 354,651). Transfection was performed with Lipofectamine™ 3,000, and mixture was added to resuspended cells before plating. Acute morphine activity was measured by plating 60 k co-transfected cells per well in serum free DMEM. 24 h later, cells were treated with or without 1 μM morphine for 4 h. For chronic morphine and withdrawal experiment, 45 k cells were plated per well in DMEM with 10% FBS and 0.3% gentamicin. 24-h later, the media and transfection mixture were removed from the cells, and 100 μL of serum free DMEM was added to each well. 6-h after media was changed to serum free, cells were treated with or without 1 μM morphine for 18-h. Morphine was diluted in serum free media and 25 μL of diluted morphine was added to each well for a final concentration of 1 μM morphine. Morphine naïve cells received 25 μL of serum free media during this time. Chronic morphine in this system was set to 18 h as it is the minimum amount of time necessary to result in cAMP overshoot following withdrawal (Xia et al., 2011). Following the 18-h chronic treatment, cells in withdrawal condition were washed 3-times with PBS to remove the morphine and begin withdrawal (Sharma et al., 1975a; Xia et al., 2011). After 4-h of stimulation, cells were lysed for 30 min using Reporter Lysis 5X Buffer (Promega, E3971), and D-Luciferin (Sigma-Aldrich, L6882) was added. Luminescence was read 5 min after luciferin addition using TECAN Infinite® M Plex, multimode microplate reader. After the 4-h stimulation of hCOs, One-Step™ Luciferase Assay reagent (BPS Bioscience, 60,690) was added to each well and incubated for 30 min followed by luminescence reading using TECAN Infinite® M Plex, multimode microplate reader.

### 2.4 IPA analysis

hCOs were treated with morphine for 30 h after which RNA was extracted. RT-qPCR was performed with a panel of 61 genes associated with transcriptional changes in response to opioids. The expression changes in the 61 opioid response related genes were calculated using the fold change values (Fold change>1.1, >1.2, and >1.5). The ingenuity pathway analysis (IPA) was used to perform bioinformatic analysis. This includes canonical pathway analysis, upstream regulator, disease and function. The annual license for IPA, was purchased from QIAGEN Inc.^1^ Canonical pathways that are significantly enriched by the differentially expressed 61 opioid response related genes in our dataset were identified by the right-tailed Fisher’s Exact Test. The test calculates a *p*-value indicating the probability of association of the canonical pathway with the dataset is due to random chance alone. The corrected p-value was obtained using the Benjamini-Hochberg method. In addition, IPA also calculated the activation z-score to infer the activation state (activation or inhibition) of the enriched pathways. Positive Z-score indicates the activation of the pathway, whereas negative Z-score indicates the inhibition. IPA’s upstream regulator analysis identifies the top enriched upstream regulators associated with the gene expression changes in the 61 opioid response related genes. The analysis also identified the number of targets of each upstream regulator that are present among the opioid response related genes and predicted the direction of change (activation or inhibition).

### 2.5 Semi-quantitative RT-PCR and real-time qPCR of *OPRM1* isoforms

Samples were harvested and RNA was extracted following instructions from New England Biolabs (NEB, T2010S). cDNA was synthesized using previously described methods (Donadoni et al., 2019, 2022). 100 ng of cDNA was used as a template for PCR reactions. OPRM1 isoforms MOR-1 and MOR-1X were amplified by PCR using primers listed in Supplementary Table S5. The remaining 7TM OPRM1 isoforms were amplified using primers previously described (Donadoni et al., 2022). Primers were designed around unique isoform exons using Integrated DNA Technology’s (IDT) PrimerQuest design tool and purchased through IDT. *β*-actin was amplified from the same set of samples as internal control. PCR products were run on 1% DNA-agarose gels and visualized by ethidium bromide.

RT-qPCR was performed to obtain a more quantitative measurement of expression levels of MOR-1 and MOR-1X. Primers and probes designed to specifically amplify MOR-1 and MOR-1X were generated and listed in Supplementary Table S5. β-actin was amplified in the same reaction, with its probe expressing the opposite fluorophore to MOR-1 or MOR-1X probe. 50 ng of RNA was used per well to conduct one-step RT-qPCR using Luna® Universal Probe One-Step RT-qPCR Kit (NEB, E3006S) following the manufacturer’s instructions.

### 2.6 BaseScope

hCOs (*N* = 3) treated with 5 μM morphine for 6-h were fixed and embedded as previously described (Donadoni et al., 2024). BaseScope staining was performed using ACD kits and probes as indicated in previous study with slight modifications(Donadoni et al., 2024). Boxall Blocking reagent was not used and sections were treated with RNAscope ™ Protease IV (ACD™, 322,336). BaseScope™ Detection Reagents v2—RED (ACD™, 323,910) was used by following the user manual of the kit. The incubation time of Amplification 7 solution was 40 min. Two custom BaseScope probes were designed and manufactured by ACD for Exon 2 (MOR) or Exon X (MOR-1X) of OPRM1 using NCBI sequences. Exon 2 was chosen to represent total MOR expression (Liu et al., 2021). The probes that were used in this assay for hybridization were the probe for Exon 2: BaseScope™ Probe BA-Hs-OPRM1-3zz-st1-C1 (ACD, 131561-C1) or MOR, probe for Exon X: BA-Hs-OPRM1-tvMOR-1X-3zz-st1-C1 (ACD, 1307961-C1) or MOR-1X, BaseScope™ Probe BA-DapB-3zz (ACD, 701011) as negative control and BaseScope™ Probe BA-Hs-PPIB-3zz (ACD, 701031) as positive control. 4–5 images/hCO were taken with a Keyence microscope and analyzed using FIJI software to separate cells from probe. Cell number was determined with FIJI and probe count was performed.

### 2.7 Immunocytochemistry

Neurons were stained for OCT4, Pax6, MAP2, and *β*-tubulin III (TUJ1) using immunocytochemistry to confirm maturation. Cells were fixed with a solution of cold ethanol/acetone (50/50) for 5 min and washed with PBS. Slides were blocked with 10% Bovine Serum Albumin (BSA) diluted in PBS for 1 h. Following blocking, slides were incubated with primary antibodies diluted in 5% BSA overnight at 4 °C on a rocker. Primary antibodies were then removed, and slides were washed 3X times with PBS. Fluorescent secondary antibodies were diluted in 5% BSA and added to slides. Slides were incubated for 1 h at room temperature with secondary, followed by 3X PBS washes. DAPI was added drop wise to slides, and slides were sealed with coverslips. Images were taken using Keyence Fluorescence microscope.

### 2.8 Antibodies and reagents

Antibodies were obtained from the following sources: anti-human OCT4 (Stemcell, 60,093), anti-Pax-6 (Biolegend, 901,301), anti-MAP2 (Cell Signaling Technology, 8707S), Anti-Tubulin Beta 3 (TUJ1) (Santa Cruz Biotechnology, sc-58,888), anti-Myc Tag (9B11) (Cell Signaling Technology, 2,276).

### 2.9 Cloning and plasmid generation

MOR-1 isoform (NCBI ref.: NM_000914) was cloned into eukaryotic expression vector pcDNA3.1 (+) at HindIII/XhoI sites and labeled as pMOR-1. MOR-1X isoform (NCBI ref.: NM_001008505) was cloned into pcDNA3.1 (+) at HindIII/XhoI sites and labeled as pMOR-1X. The inserts were created using the following primers: MOR-1/1X Fw HindIII: 5′- AATATTAAGCTTATGGACAGCAGCGCTGCC -3′, MOR-1 Rv XhoI: 5′- AATATTCTCGAGTTAGGGCAACGGAGCAGT -3′, MOR-1X Rv XhoI: 5′- AAGGATTCTCGAGTTAACTCAGGAGAAATGC -3′. Utilizing the siRNA Wizard Software from InvivoGen, a shRNA targeting MOR-1X (LV-MOR-1X shRNA), and a scrambled shRNA (LV-scrambled shRNA), were designed MOR-1X-shRNA sequence: 5′- GAGTTAAGCATGATTATTC -3′, Scrambled-shRNA sequence: 5′- GTCTTGGTATGAAACTTAA -3′. MOR-1X-shRNA and Scrambled-shRNA were cloned: into pLKO.1 - TRC cloning vector at AgeI/EcoRI sites, following the cloning protocol provided by Addgene. Oligos compatible with pLKO.1 cloning are listed in Supplementary Table S7. pLKO.1 - TRC cloning vector was a gift from David Root (Addgene, 10,878) (Moffat et al., 2006).

### 2.10 Lentiviral packaging and neuronal transductions

HEK293T cells were co-transfected with pCMV-VSV-G, psPAX2, and either pLKO.1-scrambled-shRNA or pLKO.1-MOR1-X-shRNA plasmids using Lipofectamine™ 3,000. pCMV-VSV-G was a gift from Bob Weinberg (Addgene, 8,454), psPAX2 was a gift from Didier Trono (Addgene, 12,260) (Stewart et al., 2003). Supernatant was collected 72- and 96-h post transfection. Lenti-X™ Concentrator (Takara, 631,232) was used to concentrate lentiviral vector particles from supernatants. Concentrated virus was resuspended in Opti-MEM. Lentivirus particles were quantified using Lenti-X™ qRT-PCR Titration Kit (Takara, 631,235) following manufacturer’s instructions. Transduction of neurons was performed by diluting virus in Opti-MEM containing Polybrene® (Santa Cruz Biotechnology, sc-134220) at final concentration 5 μg/mL. Media was removed from cells and lentiviral mixture was added onto the cells for 4 h, shaking the plates every 30 min. Following 4-h incubation, fresh media was added to the well. 24 h later, a full media change was performed. Transduction of hCOs was performed using TransDux™ MAX Lentivirus Transduction Reagent. hCOs were transferred to individual wells of round-bottom 96-well plates and media was removed. Lentivirus was diluted in Opti-MEM to desired MOI, and TransDux™ and MAX enhancer were added to a final concentration of 1X. 150 μL of lentiviral mix was added to each hCO containing well. hCOs were subjected to spinoculation at 1200 g for 2 h at 32 °C. The plate was then transferred to incubator for 4 h. Following 4-h incubation, hCOs were transferred to individual wells of 24-well ultra-low attachment plates with 1 mL fresh media and placed on orbital shaker.

### 2.11 Cellular plasmid transfection

Cells were plated in Poly-D-lysine (Sigma-Aldrich, P7280) coated 60 mm dishes at a density of 7.5×10^5^ cells/dish and incubated for 24 h to allow attachment. Following 24 h, cells were transfected with 5 μg plasmid using Lipofectamine™ 3,000 at a ratio of 1:2 following manufacturer’s instructions. Transfection mixture was added to cells and incubated for 24 h. After 24-h transfection, the media was aspirated, and cells were serum starved for 24 h using DMEM with 1% FBS to synchronize cells prior to morphine treatment. Cells were then treated with 1 μM morphine diluted directly into the cell culture media for 6 h. Morphine was added at 0 h and 3 h, and cells were harvested after 6 h using TrypLE™ express.

### 2.12 Statistical analysis

All the values on the graphs are presented as mean and the standard errors of the mean are reported as error bars on the histograms. Student’s *t* test and ANOVA were performed and *p* values < 0.05 were considered statistically significant. Post-hoc Fishers LSD were used with ANOVA, and samples were age matched when necessary. Normal distribution was assumed and no formal normality testing was performed due to the small sample sizes. The raw data were analyzed by GraphPad Prism version 9.4.1.

## 3 Results

### 3.1 Human cerebral organoids (hCOs) recapitulate opioid-induced cAMP signaling and withdrawal responses

**Figure 1:**
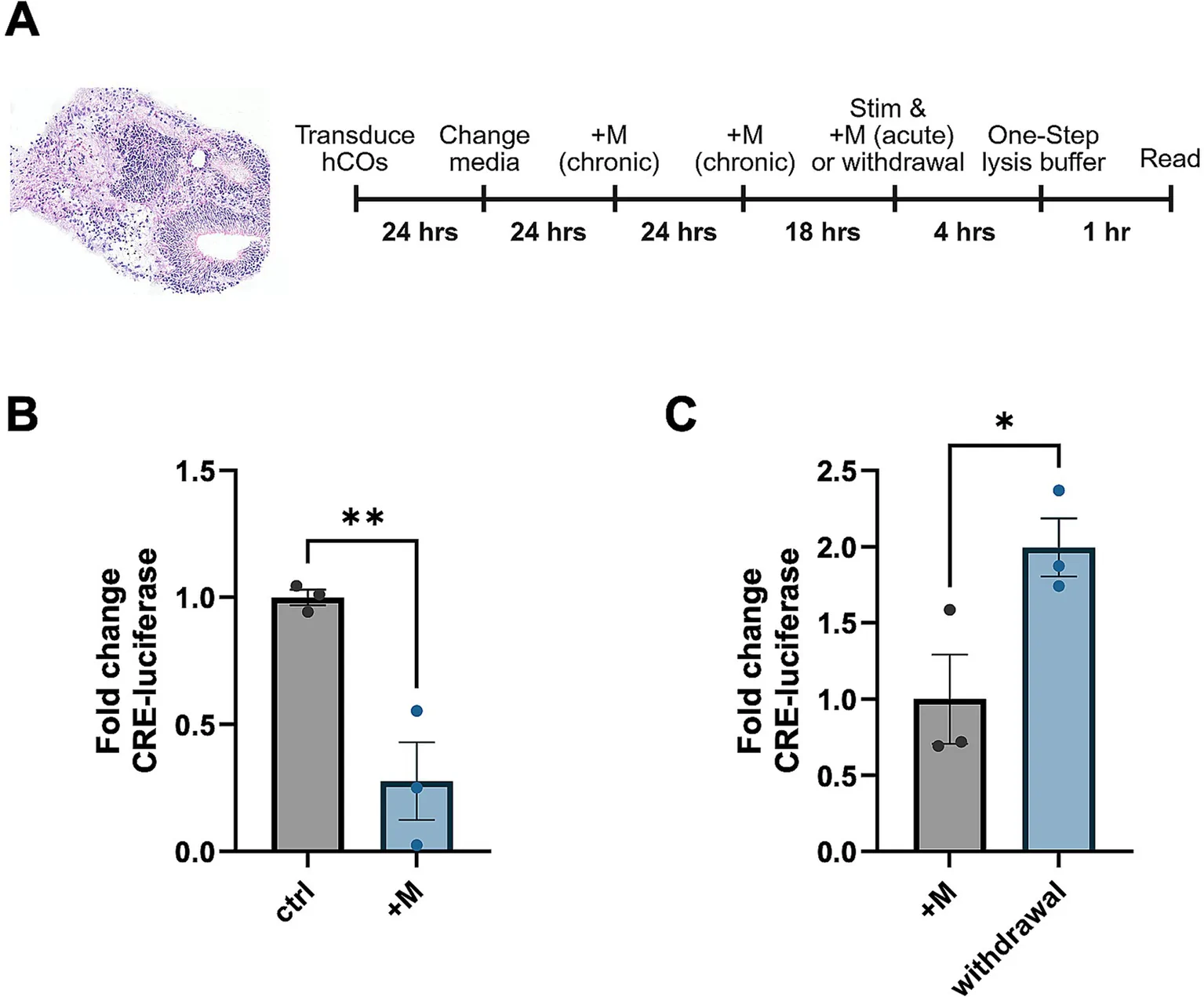
hCOs exhibit cAMP inhibition and overshoot following acute and chronic morphine treatment. **(A)** Schematic of transduction and acute and chronic morphine treatment in hCOs. **(B,C)** hCOs were treated with or without morphine (5 μM) for 4 h (**B**, acute) or 36 h (**C**, chronic). After chronic treatment, a withdrawal phase was initiated by 3x PBS washes. hCOs were stimulated with 0.5 mM IBMX, 0.5 mM Ro20-1724, and 1 μM forskolin for 4 h and CRE-luciferase expression was measured. Fold change was normalized to respective control. Significance was determined using Student *t*-test (*p* < 0.05).

hCOs were generated from hiPSCs using an unguided protocol, which allows the hCO to self-pattern into forebrain, midbrain, and hindbrain regions, resulting in a 3D recapitulation of the entire human brain *in vitro* (Lancaster et al., 2013; Zhao et al., 2022; Bellizzi et al., 2024; Donadoni et al., 2024). These hCOs were previously characterized for cell types, confirming the presence of neurons, astrocytes, oligodendrocytes, and microglia (Donadoni et al., 2024). To determine the responsivity of hCOs to morphine, we measured their cAMP signaling using transduction with a CRE promoter lentivirus as described in materials and methods. hCOs were transduced with a GFP-lentivirus and fluorescently imaged to confirm the expression of transduced lentivirus (Supplementary Figure S1). At the cellular level, a hallmark of opioid signaling is its effects on cAMP levels. Acutely, opioids inhibit adenylate cyclase (AC) and subsequently decrease cAMP levels. However, chronic opioid exposure results in a compensatory increase in AC activity, which can be seen during opioid withdrawal in a phenomenon known as cAMP overshooting. Following opioid withdrawal, there is a significant spike in cAMP levels in the cells, which has become the standard for measuring opioid dependence *in vitro.* To determine these effects in hCOs, hCOs were treated with 5 μM morphine either acutely, or chronically and subjected to withdrawal with timelines shown in Figure 1A. Following acute morphine treatment, a significant decrease in CRE-luciferase was detected, indicating a reduction in cAMP levels in the hCOs in response to morphine (Figure 1B). hCOs were then treated chronically with 5 μM morphine (Figure 1C), after which they were subjected to spontaneous withdrawal by PBS wash. This resulted in a significant increase in CRE-luciferase activity in withdrawal condition compared to chronically treated hCOs. Taken together, this demonstrates the ability of the hCOs to respond to morphine and model cellular adaptations related to opioid dependence and withdrawal.

### 3.2 Morphine modulates opioid-responsive gene networks in hCOs

**Figure 2:**
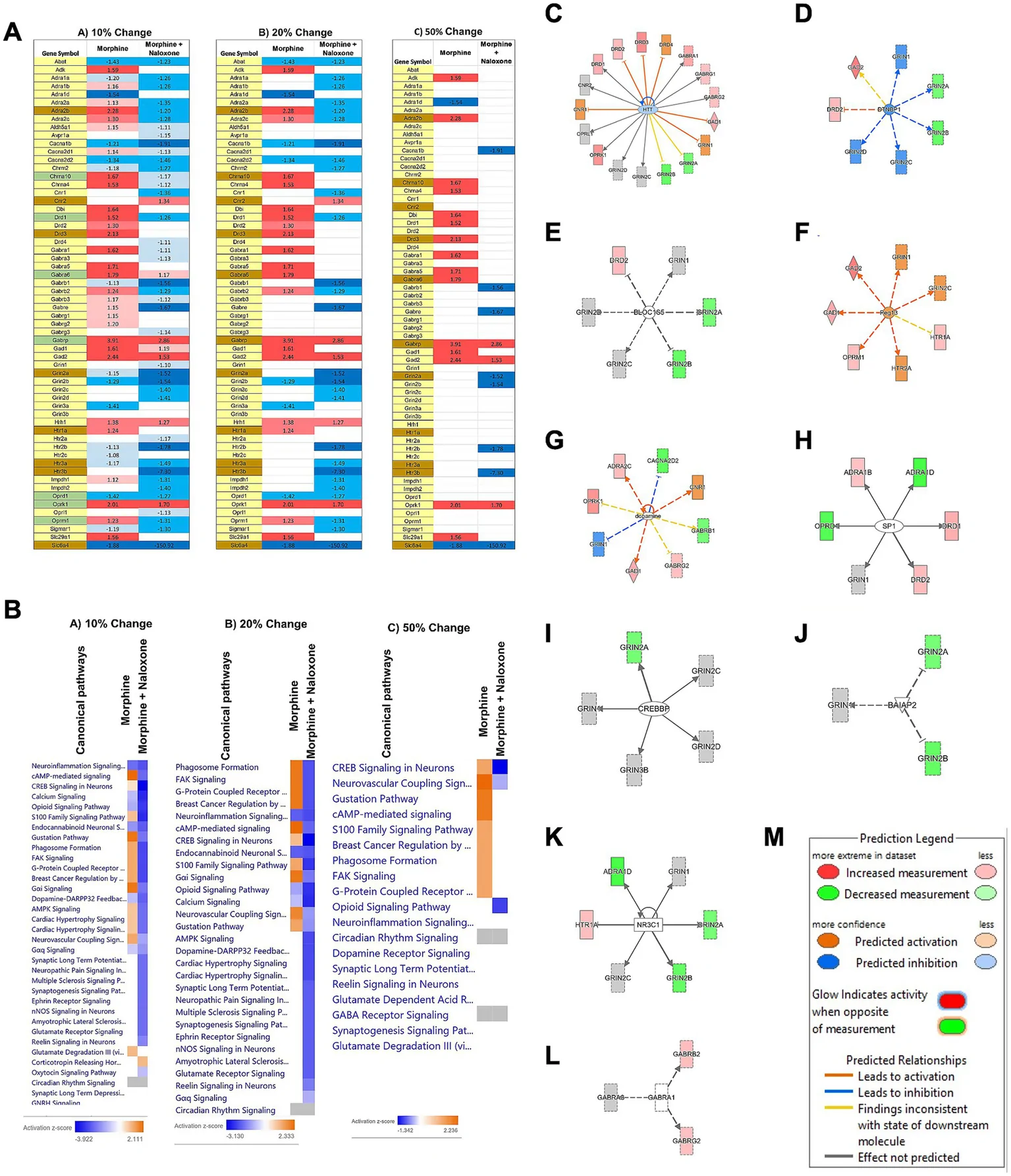
Expression changes in the 61 genes associated with opioid transcriptional responses following morphine and naloxone administration in hCOs. hCOs were treated with 5 μM morphine with or without 50 μM naloxone for 30 h. Total RNA samples were extracted, followed by RT-qPCR for a panel of 61 opioid transcriptional response related genes. **(A)** Gene expression changes were calculated using fold change values and shown as 10, 20, and 50% changes. **(B)** The DEGs were used to conduct the core analysis using IPA. Significantly (*p* < 0.05) enriched pathways associated with opioid response genes following morphine and combination morphine and naloxone treatment are shown. The activation Z-score was calculated to predict the magnitude of change in the enriched canonical pathways. **(C–M)**. Top 10 upstream regulators associated with the differentially expressed genes exhibiting 10%-fold change following morphine treatment and the predicted influence of the upstream regulators on the target genes in our dataset.

hCOs were treated with 5 μM morphine with or without 50 μM naloxone for 30 h. Total RNA samples were extracted, and RT-qPCR were performed for a panel of 61 genes associated with transcriptional changes in response to opioids. Gene expression changes were analyzed by IPA in three levels, 10, 20, and 50% change (Figure 2A). Gene expression changes were determined by normalizing expression levels in treatment groups (morphine or morphine + naloxone) to expression in untreated control samples. Genes showing 10 and 20% expression changes following morphine treatment were reversed by naloxone treatment. The genes exhibiting 50%-fold change following morphine treatment were brought to the baseline following naloxone treatment (Figure 2A). The differentially expressed genes (DEGs) were used to conduct the core analysis using IPA. Supplementary Table S1 shows the significantly (*p* < 0.05) enriched pathways associated with opioid transcriptional response related genes following morphine and combination morphine and naloxone treatment (Supplementary Table S1). The activation Z-score was then calculated to predict the magnitude of change in the enriched canonical pathways. Figure 2B shows the activation state of the enriched pathways. Pathways activated include cAMP-mediated signaling, CREB signaling in neurons, GABA receptor signaling, GPCR signaling, and Gαi signaling, which are all linked to canonical opioid signaling in the brain. Pathways activated by morphine treatment were found to be inhibited by naloxone. Supplementary Table S2 shows significantly (*p* < 0.05) enriched pathways and the top 10 upstream regulators associated with set of opioid transcriptional response related genes exhibiting fold change of 10% and above, while Supplementary Tables S3, S4 show 20 and 50%, respectively, (Supplementary Tables S2–S4). Upstream regulators identified to have fold change of 10% and above include HTT, DTNBP1, BLOC1S5, Peg13, dopamine, SP1, CREBBP, BAIAP2, NR3C1, GABRA1. Figures 2C–L shows the activation state and their influence on the target genes in the dataset of opioid transcriptional response related genes. The activation state of the upstream regulator is based on the fold change values in the dataset. IPA also predicts the influence of the upstream regulators in the activation states of the target genes in our dataset. Supplementary Figures S2, S3 shows the activation states of genes exhibiting fold change of 20 and 50%.

### 3.3 Morphine induces alternative splicing of OPRM1 and upregulates the MOR-1X isoform in hCOs

**Figure 3:**
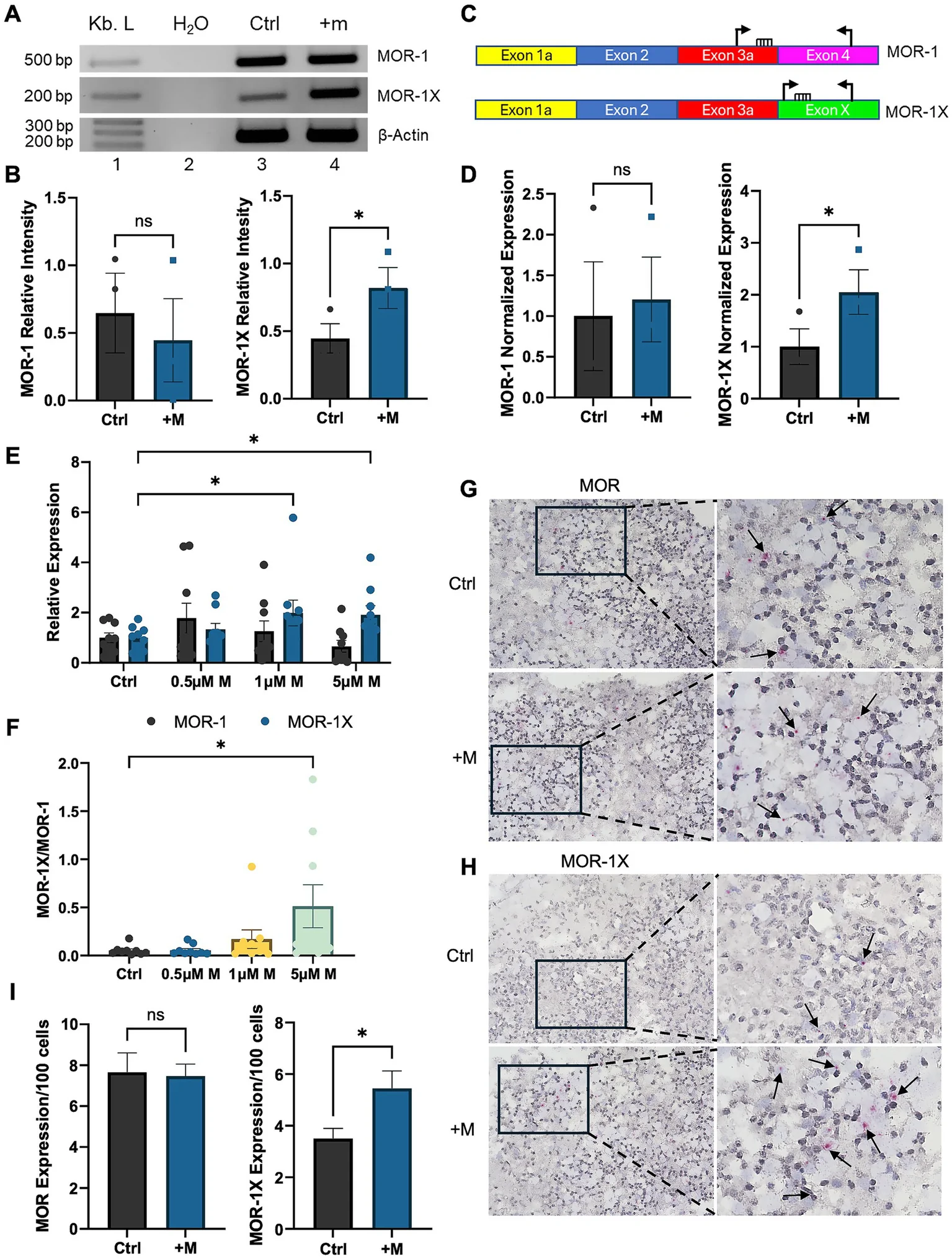
Morphine selectively induces alternative splicing of MOR-1X isoform in acute morphine treated hCOs. **(A)** hCOs were treated with 5 μM morphine for 6 h. Three hCOs were combined per case, and total RNA samples were processed by RT-PCR using unique primers for MOR-1 and MOR-1X isoforms. *β*-actin was also amplified from the same samples as internal control. **(B)** Normalized MOR-1 and MOR-1X band intensities from panel **A** were determined by Photoshop and shown as relative expression in a bar graph. **(C)** Map of unique RT-qPCR primers and probe for MOR-1 and MOR-1X isoforms. **(D)** RT-qPCRs for MOR-1 and MOR-1X from total RNA samples. Student’s *t*-tests were performed to determine significance. **(E)** hCOs were treated with morphine at concentrations of 0.5 μM, 1 μM, and 5 μM for 6 h (*N* = 9). RNA from individual hCOs was isolated and samples were processed by one-step RT-qPCR for MOR-1 and MOR-1X isoform expression, as well as β-actin as an internal control. Relative expression of MOR-1 and MOR-1X isoforms was normalized to expression in untreated hCOs. Statistics were done using two-way ANOVA. **(F)** Ratio of MOR-1X/MOR-1 expression in morphine treated hCOs. Statistics were done with a one-way ANOVA. (G, H) hCOs treated with 5 μM morphine for 6 h were fixed and processed for BaseScope to detect MOR and MOR-1X (*N* = 3). Representative 40x images of MOR **(G)** or MOR-1X **(H)** probed control and morphine treated hCOs. Probes are shown as red dots. **(I)** Expression levels of MOR and MOR-1X BaseScope images were normalized to cell number as dots/100 cells and shown as bar graphs. Student’s *t*-test was done to determine significance.

*OPRM1* pre-mRNA is known to undergo extensive alternative splicing, resulting in over 20 distinct isoforms. To determine the effect of morphine on OPRM1 isoforms in hCOs, hCOs were treated with 5 μM morphine for 6 h, after which RNA was isolated and analyzed using semi-quantitative RT-PCR for all the 11 members of the 7TM OPRM1 isoforms (Supplementary Figure S4) (*N* = 9 total, 3 pools of 3 hCOs). Morphine treatment had no effect on the expression of the canonical MOR-1 isoform and MOR-1A, MOR-1A2, MOR-1B1, MOR-1B2, MOR-1B3, MOR-1B4, MOR-1B5, MOR-1i, MOR-1Y, and MOR-1O isoforms (Supplementary Figure S5), however it resulted in a significant induction of the MOR-1X isoform (Figures 3A,B), an observation that is consistent with the SHSY5Y findings. To validate these findings in a more quantitative manner, qPCR primers and probes were designed specifically for either the MOR-1 or MOR-1X isoforms as described in materials and methods (Figure 3C, Supplementary Table S5). One step RT-qPCR was performed using RNA from the same samples used for RT-PCR, and expression levels of MOR-1 and MOR-1X were normalized to *β*-actin expression in samples. RT-qPCR confirmed that morphine had no effect on the expression levels of MOR-1, however resulted in a significant induction of MOR-1X (Figure 3D). Furthermore, these RNA samples were used for RT-qPCR of the 12 members of the serine and arginine rich splicing factors (SRSFs) to confirm that morphine alters expression of splicing regulatory genes in hCOs. Morphine exposure resulted in significant upregulation of SRSF1, 2, 4, 6, 8, 9, 11, and 12 (Supplementary Figure S6, Supplementary Table S6).

To further explore the effects of morphine treatment on MOR-1X expression, hCOs were treated with increasing concentrations of morphine (0 μM, 0.5 μM, 1 μM, and 5 μM) for 6 h, after which RNA was isolated followed by RT-qPCR for MOR-1 and MOR-1X levels (*N* = 9/concentration). A significant increase in MOR-1X levels was detected at 1 μM and 5 μM morphine in these treated hCOs, while there were no significant changes in MOR-1 levels (Figure 3E). Furthermore, the ratio of MOR-1X/MOR-1 increases with increasing doses of morphine, with a significant difference at 5 μM (Figure 3F).

BaseScope™ probes were designed with Advanced Cell Diagnostics (ACD) for *OPRM1* Exon 2 (MOR) and Exon X (MOR-1X) using their NCBI reference sequences for each exon. Exon 2 was chosen to represent total levels of MOR RNA, as exon 2 is present in all but two *OPRM1* isoforms. In contrast, Exon X is expressed exclusively in MOR-1X, thus only provides signal in the presence of this specific isoform RNA. hCOs were treated with or without 5 μM morphine for 6 h, after which they were fixed, embedded, and sliced. Sections were then probed using BaseScope™ for MOR (Figure 3G) and MOR-1X (Figure 3H). Positive probe signal is represented as red staining. Expression levels of MOR and MOR-1X were determined by number of positive signals per 100 cells (Figure 3I). Interestingly, this revealed no significant change in total levels of MOR, however there was a significant increase in MOR-1X expression in morphine treated hCOs (Figure 3I). Together, this data suggests that morphine exposure preferentially induces the MOR-1X isoform in an hCO model.

### 3.4 Morphine induces dose-dependent upregulation of MOR-1X isoform in human iPSC-derived forebrain and midbrain neurons

**Figure 4:**
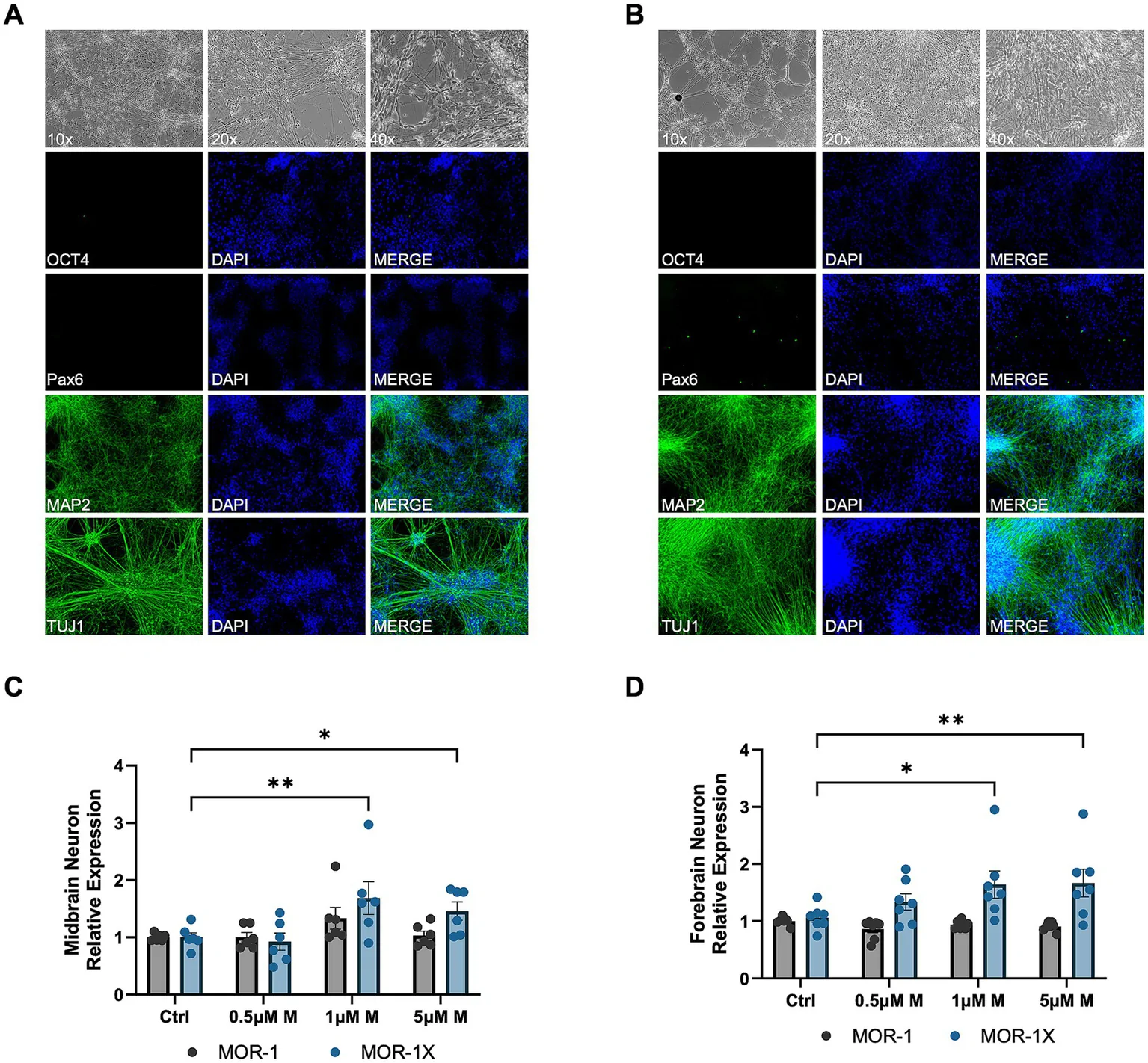
MOR-1X induction by morphine in hiPSC-derived midbrain and forebrain neurons. **(A,B)** Mature iPSC-derived midbrain **(A)** and forebrain **(B)** neurons fixed and processed by ICC for pluripotency markers OCT4 and Pax6, and neuron maturation markers MAP2 and TUJ1. (C, D) Neurons were treated with morphine at concentrations of 0.5 μM, 1 μM, and 5 μM for 6 h. RNA was isolated and samples were processed by one-step RT-qPCR for MOR-1 and MOR-1X isoform expression. Relative expressions of MOR-1 and MOR-1X isoforms in midbrain neurons **(C)** and forebrain neurons **(D)** were normalized to expression in untreated neurons. Statistics were determined using two-way ANOVA.

To measure the induction of MOR-1X by morphine specifically in neurons, iPSC derived neural progenitor cells (NPCs) were differentiated into forebrain and midbrain neurons as described in materials and methods. NPC identity was confirmed through expression of Pax6 using immunocytochemistry (Supplementary Figure S7). Maturation of these neurons was confirmed by morphology and positive staining of TUJ1 and MAP2, as well as negative staining for iPSC and NPC markers OCT4 and Pax6 to confirm lack of iPSCs (OCT4) and NPCs (Pax6) in culture (Figures 4A,B). Mature neurons were treated with 0 μM, 0.5 μM, 1 μM, or 5 μM morphine for 6 h and RNA was isolated followed by RT-qPCR for MOR-1 and MOR-1X isoforms (*N* = 9). This revealed a significant increase in MOR-1X expression at 1 μM and 5 μM morphine in both midbrain and forebrain neurons (Figures 4C,D, respectively). There was no change in MOR-1 isoform expression in either midbrain or forebrain neurons treated with any dose of morphine.

### 3.5 MOR-1X isoform drives cAMP overshoot and morphine dependence

**Figure 5:**
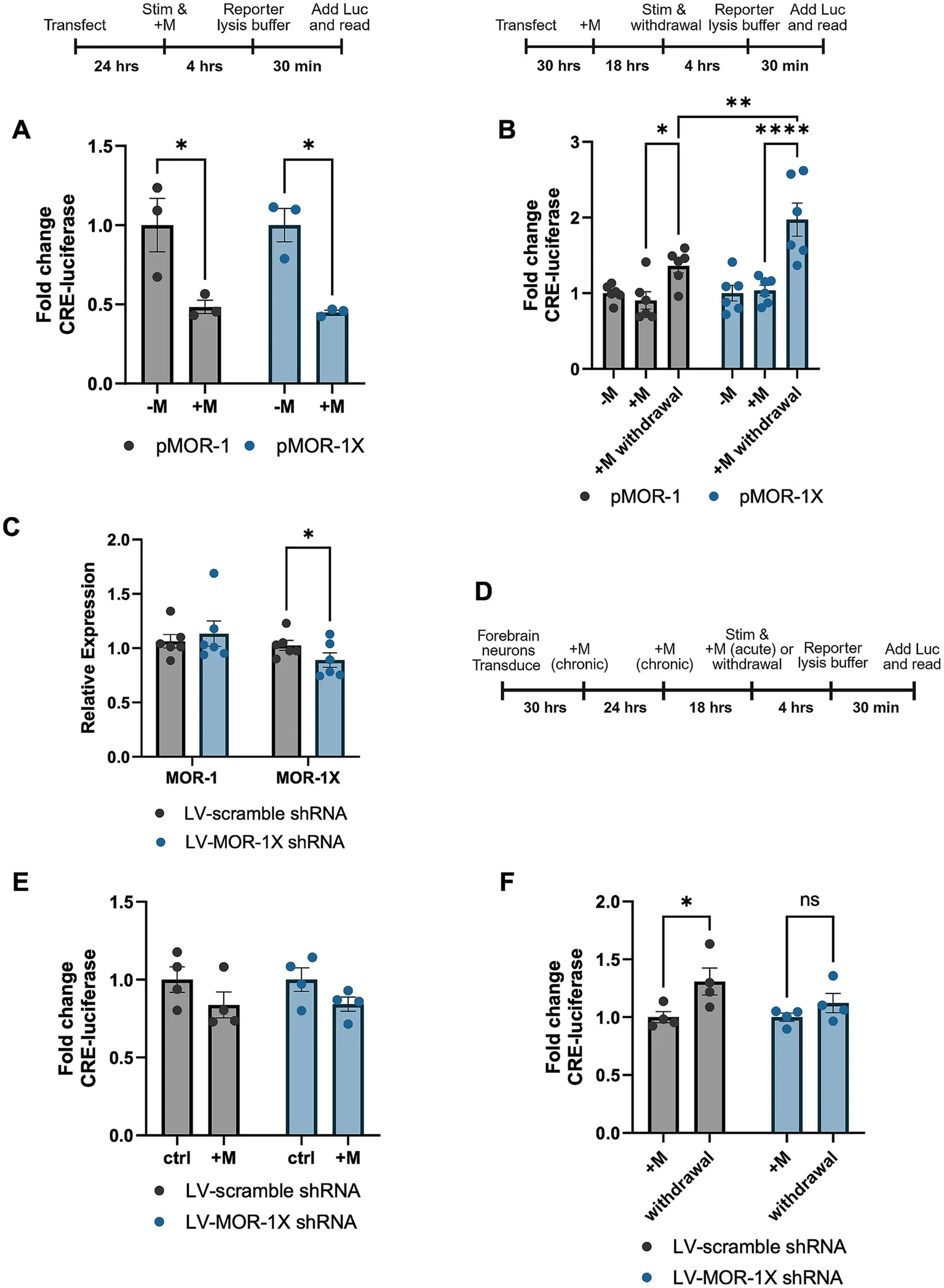
MOR-1 and MOR-1X effects on cAMP signaling. **(A,B)** HEK293 cells were transfected with CRE-luciferase plasmid and pMOR-1 or pMOR-1X and treated with or without morphine (1 μM) and stimulation mixture. **(A)** Cells were treated acutely with morphine for 4 h with stimulation mixture, after which CRE-luciferase expression was measured. **(B)** Cells were chronically treated with morphine for 18 h. After chronic treatment, a withdrawal phase was initiated, followed by the addition of stimulation mixture for 4 h, after which CRE-luciferase expression was measured. Schematic timeline of each experiment is listed above the respective bar graph. **(C)** Forebrain neurons were transduced with LV-scramble or LV-MOR-1X, after which RNA was extracted and processed by one-step RT-qPCR for MOR-1 and MOR-1X isoform expression, as well as β-actin as an internal control**. (D)** Schematic timeline of acute and chronic morphine treatments in lentiviral transduced neurons. **(E,F)** Forebrain neurons transfected with CRE-luciferase lentivirus and LV-scramble or LV-MOR-1X shRNA and were treated with or without morphine (5 μM) and stimulation mixture. **(E)** Forebrain neurons were treated acutely with morphine for 4 h with stimulation mixture, after which CRE-luciferase expression was measured. **(F)** Forebrain neurons chronically treated with morphine for 36 h. After chronic treatment, a withdrawal phase was initiated, followed by the addition of stimulation mixture for 4 h, after which CRE-luciferase expression was measured. Fold change of luminescence was normalized to respective control. Two-way ANOVA was used to determine significance.

Although cellular responses to MOR-1 activation by morphine are well-characterized, isoform-specific responses remain largely unexplored. To address this, MOR-1X effects on cAMP signaling in response to morphine was compared to the canonical MOR-1 isoform in HEK293 cells which do not express endogenous OPRM1 receptor. Cells were transfected with expression plasmids encoding either MOR-1 or MOR-1X isoforms, and isoform specificity was confirmed via RT-qPCR (Supplementary Figure S8B) and immunostaining (Supplementary Figure S8C). HEK293 cells were co-transfected with CRE-luciferase and MOR-1 or MOR-1X plasmids and treated with or without 1 μM morphine under acute or chronic/withdrawal conditions to assess isoform-specific effects on cAMP signaling (N = 6). Acute morphine treatment significantly reduced luciferase activity (*p* = 0.0008) in both MOR-1 (*p* = 0.0142) and MOR-1X (*p* = 0.0102) transfected cells, indicating that MOR-1X inhibits cAMP similarly to MOR-1 (Figure 5A). Notably, MOR-1X transfected cells exposed to chronic morphine and withdrawal exhibited a greater luciferase response compared to MOR-1 as revealed by ANOVA post-hoc (main effect interaction *p* = 0.0404, MOR-1X + withdrawal vs. MOR-1 + withdrawal *p* = 0.0035), reflecting a higher cAMP overshoot during withdrawal (Figure 5B).

An shRNA construct specifically targeting the MOR-1X isoform was generated (Supplementary Figure S9) and cloned into lentiviral vectors to investigate the effect of MOR-1X knockdown on neuronal responses to morphine, particularly during withdrawal. Validation of this shRNA’s ability to prevent the induction of MOR-1X following morphine treatment was performed (Supplementary Figure S9D). Transduction of mature forebrain neurons with either scrambled or MOR-1X specific shRNA lentivirus resulted in a significant reduction of MOR-1X expression, as measured by RT-qPCR, with no change in MOR-1 levels (*N* = 6) (Figure 5C). Mature forebrain neurons were cultured in 96-well plates following standard protocols. Upon maturation, neurons were co-transduced with CRE-luciferase lentivirus and either scrambled or MOR-1X shRNA lentivirus. Cells were subsequently treated with 5 μM morphine under acute or chronic/withdrawal conditions (*N* = 4) (Figure 5D). Acute morphine treatment inhibited cAMP, but no significant differences in CRE-luciferase activity were observed between scrambled and MOR-1X shRNA groups individually (Figure 5E). However, two-way ANOVA revealed that acute morphine had an overall significant effect on CRE-luciferase activity (*p* = 0.0498) (Figure 5E), indicating that acute cAMP inhibition occurs independently of MOR-1X knockdown. In contrast, neurons chronically treated with morphine and subjected to withdrawal displayed the expected cAMP overshoot when transduced with scrambled lentivirus (treatment *p* = 0.0166, post-hoc scrambled *p* = 0.0155) (Figure 5F). More interestingly, this overshoot was significantly attenuated by MOR-1X knockdown (*p* = 0.2865), suggesting that MOR-1X induction may be necessary for cellular adaptations associated with morphine dependence related signaling.

## 4 Discussion

Despite decades of research, the precise mechanisms underlying opioid dependence and addiction have yet to be uncovered (Kosten and George, 2002; Volkow and Blanco, 2021; Dydyk et al., 2025). This is in part due to lack of comprehensive models to study opioid signaling in a human based system. Mechanisms of opioid signaling, while having some homology among organisms, remain unique to each (Stevens, 2009; Pasternak and Pan, 2013; Liu et al., 2021). Sequences of opioid receptors between rats, mice, and humans are vastly different. Different splice variants and SNPs have been identified between each organism, all which make it difficult to identify and target human specific alterations to identify underlying causes of opioid dependence (Peciña et al., 2015; Liu et al., 2021; Piltonen et al., 2021). Previously, research on human specific changes have relied on cell lines and post-mortem brain tissue. However, since the establishment of iPSC derived cerebral organoids and other primary cells, there is access to more complex and comprehensive models of the human brain (Lancaster et al., 2013; Fernandes et al., 2022; Coronel et al., 2025). These systems have been used in various extents to model opioid signaling, however majority of studies focus on effects of opioids on neurodevelopment of these hCOs and precise functional readouts and responses to opioids have not been completely established (Strong et al., 2023; Ho et al., 2024). The result is a gap in the functionality and ability of these hCOs to act as a representative model of opioid signaling in the human brain in an *in vitro* environment.

Here, we show that iPSC-derived hCOs are a functional model of morphine activity *in vitro*. These hCOs show inhibition of cAMP levels during acute morphine treatment, as well as canonical overshoot following withdrawal, establishing a functional readout of opioid signaling in these hCOs (Bie et al., 2005; Xia et al., 2011; Fernandes et al., 2022; Swingler et al., 2025). Canonical opioid function in these hCOs is confirmed with IPA analysis following screen of 61 opioid transcriptional response related genes. Critical opioid related pathways such as cAMP and GABA associated pathways are seen to be upregulated by morphine. Pathways activated by morphine were blocked by naloxone, showing that the activation is due to morphine activity. Together, this establishes a powerful model for exploring opioid effects on the human brain in a complex *in vitro* system, allowing us to explore human specific signaling events that results in the formation of opioid dependence. While these hCOs may now be used as a functional model, it is important to note that they lack peripheral immune components and vascular elements, which are important aspects of many disorders, including OUD (McCarthy et al., 2001; Watkins et al., 2007; Plein and Rittner, 2018). As a result, further improvements of hCOs as a model are critical to expand on our understanding of the human brain.

Limited studies exist studying alternatively spliced isoforms of *OPRM1*, with even less studying the role these isoforms may have in opioid signaling, adaptation and dependence. Our results show the preferential induction of the MOR-1X isoform in response to morphine in hCOs, as well as midbrain and forebrain derived neurons; an effect that is morphine dose dependent. This data, taken with our previously published findings, shows the induction of MOR-1X specifically in a myriad of systems, thus validating the idea that *OPRM1* alternative splicing is affected by morphine exposure (Regan et al., 2016; Donadoni et al., 2022).

The MOR-1X isoform has an extended C-terminal compared to MOR-1, and phosphorylation site prediction revealed MOR-1X contains five additional phosphorylation sites, which involve PKA, DNAPK, cdc2, and unspecified (Supplementary Figure S4B) (Blom et al., 2004). These additional phosphorylation sites may result in different downstream pathway activation following agonism of the receptor. To explore the specific role MOR-1X has in opioid signaling and dependence related adaptations, we measured cAMP pathway activation. Effects of MOR activation by opioids on cAMP pathway activity is widely characterized and is the most accepted way to measure opioid dependence signaling *in vitro* (Nestler and Aghajanian, 1997; Al-Hasani and Bruchas, 2011; Xia et al., 2011; Nestler, 2012; Vandeputte et al., 2022). Opioids such as morphine are known to acutely inhibit AC following activation. However, chronic exposure to an opioid results in a compensatory increase in AC activity (Nestler and Aghajanian, 1997; Bie et al., 2005; Al-Hasani and Bruchas, 2011; Xia et al., 2011; Swingler et al., 2025). This dependence can be seen as a spike in cAMP levels following withdrawal; a phenomenon known as cAMP overshoot (Sharma et al., 1975b, 1975a; Xia et al., 2011). Our results show that MOR-1X inhibits AC in the same manner as MOR-1, however results in significantly higher levels of withdrawal in the same system. Additionally, knocking down MOR-1X expression by shRNA in neurons had no effect on inhibition of AC, however it prevented cAMP overshoot following withdrawal. This suggests that MOR-1X may play a crucial role in morphine dependence related signaling and withdrawal associated cAMP signaling pathways *in vitro*. Additionally, this shows the potential of shRNA to target specific isoforms.

Altogether, this data characterizes canonical morphine signaling in hCOs, as well as establishes a functional readout of acute and chronic/withdrawal signaling in these hCOs. This opens the door to using hCOs to further understand molecular signaling underlying opioid dependence in an *in vitro* model of the human brain. In addition, our results demonstrate that the inducible OPRM1 isoform MOR-1X functions differently than the canonically studied isoform, and it may play a key role in the development of opioid dependence signaling through effects on the cAMP pathway. Further exploration of MOR-1X specific signaling may result in identification of novel targets and pathways involved in dependence related signaling, and thus, new treatment options.

## Data Availability

The original contributions presented in this study are included in the article and its Supplementary Material. Further inquiries can be directed to the corresponding authors.

## Footnotes

1 https://www.qiagenbioinformatics.com/products/ingenuitypathway-analysis; Germantown, MD, USA.

## References

[ref1] Abrimian A. Kraft T. Pan Y.-X. (2021). Endogenous opioid peptides and alternatively spliced mu opioid receptor seven transmembrane carboxyl-terminal variants. Int. J. Mol. Sci. 22:3779. doi: 10.3390/ijms22073779, 33917474 PMC8038826

[ref2] Al-Hasani R. Bruchas M. R. (2011). Molecular mechanisms of opioid receptor-dependent signaling and behavior. Anesthesiology 115, 1363–1381. doi: 10.1097/ALN.0b013e318238bba6, 22020140 PMC3698859

[ref3] Bellizzi A. Çakır S. Donadoni M. Sariyer R. Liao S. Liu H. . (2024). Suppression of HSV-1 infection and viral reactivation by CRISPR-Cas9 gene editing in 2D and 3D culture models. Mol. Ther. Nucleic Acids 35:102282. doi: 10.1016/j.omtn.2024.102282, 39176174 PMC11339036

[ref4] Bie B. Peng Y. Zhang Y. Pan Z. Z. (2005). cAMP-mediated mechanisms for pain sensitization during opioid withdrawal. J. Neurosci. 25, 3824–3832. doi: 10.1523/JNEUROSCI.5010-04.2005, 15829634 PMC6724939

[ref5] Birtele M. Lancaster M. Quadrato G. (2025). Modelling human brain development and disease with organoids. Nat. Rev. Mol. Cell Biol. 26, 389–412. doi: 10.1038/s41580-024-00804-1, 39668188

[ref6] Blom N. Sicheritz-Pontén T. Gupta R. Gammeltoft S. Brunak S. (2004). Prediction of post-translational glycosylation and phosphorylation of proteins from the amino acid sequence. Proteomics 4, 1633–1649. doi: 10.1002/pmic.200300771, 15174133

[ref7] Brown T. G. Xu J. Hurd Y. L. Pan Y.-X. (2022). Dysregulated expression of the alternatively spliced variant mRNAs of the mu opioid receptor gene, OPRM1, in the medial prefrontal cortex of male human heroin abusers and heroin self-administering male rats. J. Neurosci. Res. 100, 35–47. doi: 10.1002/jnr.24640, 32506472 PMC8143898

[ref8] CDC (2025) Understanding the Opioid Overdose Epidemic Overdose Prev. Available online at: https://www.cdc.gov/overdose-prevention/about/understanding-the-opioid-overdose-epidemic.html (Accessed October 30, 2025).

[ref9] Chakrabarti S. Liu N.-J. Gintzler A. R. (2020). Phosphorylation of unique C-terminal sites of the mu-opioid receptor variants 1B2 and 1C1 influences their Gs association following chronic morphine. J. Neurochem. 152, 449–467. doi: 10.1111/jnc.14863, 31479519 PMC7042086

[ref10] Coronel R. González-Sastre R. Mateos-Martínez P. Maeso L. Llorente-Beneyto E. Martín-Benito S. . (2025). Human cerebral organoids: complex, versatile, and human-relevant models of neural development and brain diseases. Neural Regen. Res. 21, 837–854. doi: 10.4103/NRR.NRR-D-24-01639, 40364645 PMC12296421

[ref11] Donadoni M. Cakir S. Bellizzi A. Swingler M. Sariyer I. K. (2024). Modeling HIV-1 infection and NeuroHIV in hiPSCs-derived cerebral organoid cultures. J. Neurovirol. 30, 362–379. doi: 10.1007/s13365-024-01204-z, 38600307 PMC11464638

[ref12] Donadoni M. Cicalese S. Sarkar D. K. Chang S. L. Sariyer I. K. (2019). Alcohol exposure alters pre-mRNA splicing of antiapoptotic mcl-1L isoform and induces apoptosis in neural progenitors and immature neurons. Cell Death Dis. 10:447. doi: 10.1038/s41419-019-1673-3, 31171771 PMC6554352

[ref13] Donadoni M. Huang W. Yarandi S. S. Burdo T. H. Chang S. L. Sariyer I. K. (2022). Modulation of OPRM1 alternative splicing by morphine and HIV–1 Nef. J. Neuroimmune Pharmacol. 17, 277–288. doi: 10.1007/s11481-021-10009-4, 34420144 PMC8859008

[ref14] Dowell D. (2024). Treatment for opioid use disorder: population estimates — United States, 2022. MMWR Morb. Mortal Wkly. Rep. 73, 567–574. doi: 10.15585/mmwr.mm7325a1, 38935567 PMC11254342

[ref15] Dydyk A. M. Jain N. K. Gupta M. (2025). Opioid use disorder: evaluation and management. Available online at: http://www.ncbi.nlm.nih.gov/books/NBK553166/ (Accessed November 16, 2025).

[ref16] El Kouhen R. Burd A. L. Erickson-Herbrandson L. J. Chang C. Y. Law P. Y. Loh H. H. (2001). Phosphorylation of Ser363, Thr370, and Ser375 residues within the carboxyl tail differentially regulates mu-opioid receptor internalization. J. Biol. Chem. 276, 12774–12780. doi: 10.1074/jbc.M009571200, 11278523

[ref17] Fernandes A. M. Campos J. Silva D. Barata Antunes S. Lima R. Coelho C. . (2022). Cerebral organoids to study central mechanisms of pain: the effect of stem cell Secretome on opioid receptors and neuroplasticity. Stem Cells Dev. 31, 641–657. doi: 10.1089/scd.2022.0116, 36082997

[ref18] Gris P. Gauthier J. Cheng P. Gibson D. G. Gris D. Laur O. . (2010). A novel alternatively spliced isoform of the mu-opioid receptor: functional antagonism. Mol. Pain 6:33. doi: 10.1186/1744-8069-6-33, 20525224 PMC2894766

[ref19] Herman T. F. Cascella M. Muzio M. R. (2024). Mu Receptors. Available online at: http://www.ncbi.nlm.nih.gov/books/NBK551554/ (Accessed October 22, 2024).

[ref20] Ho M.-F. Zhang C. Moon I. Zhu X. Coombes B. J. Biernacka J. . (2024). Single cell transcriptomics reveals distinct transcriptional responses to oxycodone and buprenorphine by iPSC-derived brain organoids from patients with opioid use disorder. Mol. Psychiatry 29, 1636–1646. doi: 10.1038/s41380-022-01837-8, 36302966 PMC10588459

[ref21] Kim H. S. Xiao Y. Chen X. He S. Im J. Willner M. J. . (2024). Chronic opioid treatment arrests neurodevelopment and alters synaptic activity in human midbrain organoids. Adv. Sci. 11:e2400847. doi: 10.1002/advs.202400847, 38549185 PMC11151039

[ref22] Klein A. H. Alam S. M. S. Johnson K. Kriner C. Beck B. Nelson B. . (2026). Inhibition of adenylyl cyclase 1 or exchange protein activated by cAMP restores ATP-sensitive potassium channel activity after chronic opioid exposure. Br. J. Pharmacol. 183, 3706–3722. doi: 10.1111/bph.70417, 41946477 PMC13208457

[ref23] Koch T. Schulz S. Pfeiffer M. Klutzny M. Schröder H. Kahl E. . (2001). C-terminal splice variants of the mouse mu-opioid receptor differ in morphine-induced internalization and receptor resensitization. J. Biol. Chem. 276, 31408–31414. doi: 10.1074/jbc.M100305200 11359768

[ref24] Kosten T. R. George T. P. (2002). The neurobiology of opioid dependence: implications for treatment. Sci. Pract. Perspect. 1, 13–20. doi: 10.1151/spp021113, 18567959 PMC2851054

[ref25] Lancaster M. A. Renner M. Martin C.-A. Wenzel D. Bicknell L. S. Hurles M. E. . (2013). Cerebral organoids model human brain development and microcephaly. Nature 501, 373–379. doi: 10.1038/nature12517, 23995685 PMC3817409

[ref26] Liu S. Kang W.-J. Abrimian A. Xu J. Cartegni L. Majumdar S. . (2021). Alternative pre-mRNA splicing of the mu opioid receptor gene, OPRM1: insight into complex mu opioid actions. Biomolecules 11:1525. doi: 10.3390/biom11101525, 34680158 PMC8534031

[ref27] Lu X. Yang J. Xiang Y. (2022). Modeling human neurodevelopmental diseases with brain organoids. Cell Regen. 11:1. doi: 10.1186/s13619-021-00103-6, 34982276 PMC8727646

[ref28] McCarthy L. Wetzel M. Sliker J. K. Eisenstein T. K. Rogers T. J. (2001). Opioids, opioid receptors, and the immune response. Drug Alcohol Depend. 62, 111–123. doi: 10.1016/s0376-8716(00)00181-2, 11245967

[ref29] Moffat J. Grueneberg D. A. Yang X. Kim S. Y. Kloepfer A. M. Hinkle G. . (2006). A lentiviral RNAi library for human and mouse genes applied to an arrayed viral high-content screen. Cell 124, 1283–1298. doi: 10.1016/j.cell.2006.01.040, 16564017

[ref30] Muntean B. S. Dao M. T. Martemyanov K. A. (2019). Allostatic changes in the cAMP system drive opioid-induced adaptation in striatal dopamine signaling. Cell Rep. 29, 946–960.e2. doi: 10.1016/j.celrep.2019.09.034, 31644915 PMC6871051

[ref31] Nestler E. J. (2012). Transcriptional mechanisms of drug addiction. Clin. Psychopharmacol. Neurosci. 10, 136–143. doi: 10.9758/cpn.2012.10.3.136, 23430970 PMC3569166

[ref32] Nestler E. J. Aghajanian G. K. (1997). Molecular and cellular basis of addiction. Science 278, 58–63. doi: 10.1126/science.278.5335.58, 9311927

[ref33] Pan Y. X. Xu J. Bolan E. Abbadie C. Chang A. Zuckerman A. . (1999). Identification and characterization of three new alternatively spliced mu-opioid receptor isoforms. Mol. Pharmacol. 56, 396–403. doi: 10.1124/mol.56.2.396 10419560

[ref34] Pan L. Xu J. Yu R. Xu M.-M. Pan Y.-X. Pasternak G. W. (2005). Identification and characterization of six new alternatively spliced variants of the human mu opioid receptor gene, Oprm. Neuroscience 133, 209–220. doi: 10.1016/j.neuroscience.2004.12.033, 15893644

[ref35] Pasternak G. W. Childers S. R. Pan Y.-X. (2020). “Emerging insights into mu opioid pharmacology,” in Substance Use Disorders: From Etiology to Treatment, eds. Nader M. A. Hurd Y. L. (Cham: Springer International Publishing), 89–125. 10.1007/164_2019_270 31598835

[ref36] Pasternak G. W. Pan Y.-X. (2013). Mu opioids and their receptors: evolution of a concept. Pharmacol. Rev. 65, 1257–1317. doi: 10.1124/pr.112.007138, 24076545 PMC3799236

[ref37] Peciña M. Love T. Stohler C. S. Goldman D. Zubieta J.-K. (2015). Effects of the mu opioid receptor polymorphism (OPRM1 A118G) on pain regulation, placebo effects and associated personality trait measures. Neuropsychopharmacology 40, 957–965. doi: 10.1038/npp.2014.272, 25308352 PMC4330509

[ref38] Piltonen M. Krokhotin A. Parisien M. Bérubé P. Djambazian H. Sladek R. . (2021). Alternative splicing of opioid receptor genes shows a conserved pattern for 6TM receptor variants. Cell. Mol. Neurobiol. 41, 1039–1055. doi: 10.1007/s10571-020-00971-7, 33010019 PMC8159799

[ref39] Plein L. M. Rittner H. L. (2018). Opioids and the immune system - friend or foe. Br. J. Pharmacol. 175, 2717–2725. doi: 10.1111/bph.13750, 28213891 PMC6016673

[ref40] Regan P. M. Sariyer I. K. Langford T. D. Datta P. K. Khalili K. (2016). Morphine-induced MOR-1X and ASF/SF2 expressions are independent of transcriptional regulation: implications for MOR-1X signaling. J. Cell. Physiol. 231, 1542–1553. doi: 10.1002/jcp.25246, 26553431 PMC4801751

[ref41] Sharma S. K. Klee W. A. Nirenberg M. (1975a). Dual regulation of adenylate cyclase accounts for narcotic dependence and tolerance. Proc. Natl. Acad. Sci. USA 72, 3092–3096. doi: 10.1073/pnas.72.8.3092, 1059094 PMC432926

[ref42] Sharma S. K. Klee W. A. Nirenberg M. (1977). Opiate-dependent modulation of adenylate cyclase. Proc. Natl. Acad. Sci. USA 74, 3365–3369. doi: 10.1073/pnas.74.8.3365, 269396 PMC431562

[ref43] Sharma S. K. Nirenberg M. Klee W. A. (1975b). Morphine receptors as regulators of adenylate cyclase activity. Proc. Natl. Acad. Sci. USA 72, 590–594. doi: 10.1073/pnas.72.2.590, 1054841 PMC432359

[ref44] Stevens C. W. (2009). The evolution of vertebrate opioid receptors. Front. Biosci. 14, 1247–1269. doi: 10.2741/3306, 19273128 PMC3070387

[ref45] Stewart S. A. Dykxhoorn D. M. Palliser D. Mizuno H. Yu E. Y. An D. S. . (2003). Lentivirus-delivered stable gene silencing by RNAi in primary cells. RNA N. Y. 9, 493–501. doi: 10.1261/rna.2192803, 12649500 PMC1370415

[ref46] Strong C. E. Zhang J. Carrasco M. Kundu S. Boutin M. Vishwasrao H. D. . (2023). Functional brain region-specific neural spheroids for modeling neurological diseases and therapeutics screening. Commun. Biol. 6:1211. doi: 10.1038/s42003-023-05582-8, 38017066 PMC10684574

[ref47] Swingler M. Donadoni M. Unterwald E. M. Maggirwar S. B. Sariyer I. K. (2025). Molecular and cellular basis of mu-opioid receptor signaling: mechanisms underlying tolerance and dependence development. Front. Neurosci. 19:1597922. doi: 10.3389/fnins.2025.1597922, 40630856 PMC12234547

[ref48] Valentino R. J. Volkow N. D. (2018). Untangling the complexity of opioid receptor function. Neuropsychopharmacol. Off. Publ. Am. Coll. Neuropsychopharmacol. 43, 2514–2520. doi: 10.1038/s41386-018-0225-3, 30250308 PMC6224460

[ref49] Vandeputte M. M. Vasudevan L. Stove C. P. (2022). *In vitro* functional assays as a tool to study new synthetic opioids at the μ-opioid receptor: potential, pitfalls and progress. Pharmacol. Ther. 235:108161. doi: 10.1016/j.pharmthera.2022.108161, 35183593

[ref50] Volkow N. D. Blanco C. (2021). The changing opioid crisis: development, challenges and opportunities. Mol. Psychiatry 26, 218–233. doi: 10.1038/s41380-020-0661-4, 32020048 PMC7398847

[ref51] Watkins L. R. Hutchinson M. R. Milligan E. D. Maier S. F. (2007). “Listening” and “talking” to neurons: implications of immune activation for pain control and increasing the efficacy of opioids. Brain Res. Rev. 56, 148–169. doi: 10.1016/j.brainresrev.2007.06.006, 17706291 PMC2245863

[ref52] Williams J. T. Ingram S. L. Henderson G. Chavkin C. von Zastrow M. Schulz S. . (2013). Regulation of μ-opioid receptors: desensitization, phosphorylation, internalization, and tolerance. Pharmacol. Rev. 65, 223–254. doi: 10.1124/pr.112.005942, 23321159 PMC3565916

[ref53] Xia M. Guo V. Huang R. Shahane S. A. Austin C. P. Nirenberg M. . (2011). Inhibition of morphine-induced cAMP overshoot: a cell-based assay model in a high-throughput format. Cell. Mol. Neurobiol. 31, 901–907. doi: 10.1007/s10571-011-9689-y, 21598037 PMC3146558

[ref54] Zhao Z. Chen X. Dowbaj A. M. Sljukic A. Bratlie K. Lin L. . (2022). Organoids. Nat. Rev. Methods Primers 2:94. doi: 10.1038/s43586-022-00174-y, 37325195 PMC10270325

