# Supplementary material for: Morphine-induced μ-opioid receptor isoform MOR-1X amplifies dependence-related signaling
Source: Front Neurosci. 2026 Aug 19;20:1907881. doi: 10.3389/fnins.2026.1907881 (PMC13534043; doi:10.3389/fnins.2026.1907881)
Supplement: Supplementary file 1 [file Data_Sheet_1.docx]

**Supplement table 1** shows the significantly (P<0.05) enriched pathways associated with opioid transcriptional response related genes following morphine and combination morphine and naloxone treatment. The activation Z-score was then calculated to predict the magnitude of change in the enriched canonical pathways. **Figure 1** shows the activation state of the enriched pathways. Pathways activated by morphine treatment were found to be inhibited by naloxone.

**Supplement table 2A** Top upstream regulators associated with the differentially expressed genes, exhibiting 10%-fold change following morphine treatment. **2B.** shows the top 10 upstream regulators associated with the set of opioid transcriptional response related genes exhibiting fold change of 10% and above.

**Supplement table 3A** Top upstream regulators associated with the differentially expressed genes, exhibiting 20%-fold change following morphine treatment. **3B.** shows the top 10 upstream regulators associated with the set of opioid transcriptional response related genes exhibiting fold change of 20% and above.

**Supplement table 4A** Top upstream regulators associated with the differentially expressed genes, exhibiting 50%-fold change following morphine treatment. **4B.** shows the top 10 upstream regulators associated with the set of opioid transcriptional response related genes exhibiting fold change of 50% and above.

**Supplement table 5.** RT-PCR and RT-qPCR primers and probes for MOR-1, MOR-1X, and B-actin.

**Supplement table 6**. RT-qPCR primers for SRSF1-12.

**Supplement table 7**. Primers for generation of Exon X scramble and shRNA sequences cloned into plasmid.

**Supplement figure 1.** 10x Phase contrast and GFP images of mature hCO transduced with GFP expressing lentivirus (BPS biosciences) using transdux max kit. Z-stack images were merged and overlayed to show transducibility of hCOs.

**Supplement figure 2.** Top 10 upstream regulators associated with the differentially expressed genes (exhibiting 20% fold change) following morphine treatment and the predicted influence of the upstream regulators on the target genes in our dataset. A represents the most differentially expressed and J represents the least differentially expressed.

**Supplement figure 3.** Top 10 upstream regulators associated with the differentially expressed genes (exhibiting 50% fold change) following morphine treatment and the predicted influence of the upstream regulators on the target genes in our dataset. A represents the most differentially expressed and J represents the least differentially expressed.

**Supplement figure 4. A.** OPRM1 isoform PCR primer design. Map of all OPRM1 and all 7TM isoforms with their exon compositions. Each isoform has the location of the forward and reverse primer used for PCR marked, as well as the expected amplicon size in base pairs (bp). **B.** Predicted phosphorylation sites of MOR-1 and MOR-1X C-terminal tails. Prediction was done using NetPhos.

**Supplement figure 5.** hCOs were treated with 5μM morphine for 6 hours. Three hCOs were combined and RNA was extracted. CDNA was made from this RNA, followed by RT-PCR for each 7TM OPRM1 isoform. Fig 5 shows amplification of all 7TM isoforms by PCR. Representative of N=3 cases.

**Supplement figure 6.** Using RNA from hCOs treated with 5μM morphine for 6 hours, **A.** one step RT-qPCR for the 12 RNA splicing proteins serine-arginine rich splicing factors (SRSFs) was performed using 50ng RNA. Data is graphed as Ratio to b-actin and statistics were performed using one-way ANOVA. Primers of each SRSF protein are listed as supplemental table 6.

**Supplement figure 7.** hiPSCs and hNPCs were fixed and stained by ICC with neural progenitor marker Pax6 to confirm differentiation. DAPI was used to stain nuclei. Representative 20X magnification images are shown.

**Supplement figure 8.** Validation of MOR plasmids. **A.** Maps of pcDNA3.1 plasmids pMOR-1 and pMOR-1X. **B.** RT-qPCR of pcDNA6 and pcDNA3.1 MOR-1 and MOR-1X plasmids using specific primers and probes for each. Data normalized as ratio to B-actin. **C.** HEK293 cells were transfected with pcDNA6 MOR-1 or MOR-1X plasmids. Cells were fixed and stained with Myc-tag antibody using ICC to confirm expression of protein.

**Supplement figure 9.** Validation of shRNA. **A.** Map of pLKO.1 plasmid with Exon X scramble sequence. **B.** Exon map of MOR-1X with location of designed shRNA construct. **C.** Map of pLKO.1 plasmid with Exon X shRNA sequence. These plasmids were used to generate lentiviral constructs ultimately used in experiments. **D.** SK-N-SH were plated in 12-well plates and transduced with LV-scramble or LV-shRNA and treated with or without 5µM morphine for 6 hours. Following treatment, cells were collected and RNA was extracted. RT-qPCR for MOR-1X was performed, and samples were normalized to β-actin. Data is graphed as Normalized expression to their respective control, and statistics were performed using two-way ANOVA in prism with Fisher’s LSD post-hoc test.
